# Toward Naturalistic Neuroscience of Navigation: Opportunities in Coral Reef Fish

**DOI:** 10.3389/fncir.2022.895381

**Published:** 2022-07-05

**Authors:** Shachar Givon, Renanel Pickholtz, Eliezer Y. Pickholtz, Ohad Ben-Shahar, Moshe Kiflawi, Ronen Segev

**Affiliations:** ^1^Department of Life Sciences, Ben-Gurion University of the Negev, Beersheba, Israel; ^2^Zlotowski Center for Neuroscience, Ben-Gurion University of the Negev, Beersheba, Israel; ^3^School of Zoology, George S. Wise Faculty of Life Sciences, Tel Aviv University, Tel Aviv, Israel; ^4^The Interuniversity Institute for Marine Sciences, Eilat, Israel; ^5^Independent Researcher, East Brunswick, NJ, United States; ^6^Department of Computer Science, Ben-Gurion University of the Negev, Beersheba, Israel; ^7^Department of Biomedical Engineering, Ben-Gurion University of the Negev, Beersheba, Israel

**Keywords:** navigation, electrophysiology, rabbitfish, coral reef, *Siganus rivulatus*

## Abstract

The ability to navigate in the world is crucial to many species. One of the most fundamental unresolved issues in understanding animal navigation is how the brain represents spatial information. Although navigation has been studied extensively in many taxa, the key efforts to determine the neural basis of navigation have focused on mammals, usually in lab experiments, where the allocated space is typically very small; e.g., up to one order of magnitude the size of the animal, is limited by artificial walls, and contains only a few objects. This type of setting is vastly different from the habitat of animals in the wild, which is open in many cases and is virtually limitless in size compared to its inhabitants. Thus, a fundamental open question in animal navigation is whether small-scale, spatially confined, and artificially crafted lab experiments indeed reveal how navigation is enacted in the real world. This question is difficult to study given the technical problems associated with *in vivo* electrophysiology in natural settings. Here, we argue that these difficulties can be overcome by implementing state of the art technology when studying the rivulated rabbitfish, *Siganus rivulatus* as the model animal. As a first step toward this goal, using acoustic tracking of the reef, we demonstrate that individual *S. rivulatus* have a defined home range of about 200 m in length, from which they seldom venture. They repeatedly visit the same areas and return to the same sleeping grounds, thus providing evidence for their ability to navigate in the reef environment. Using a clustering algorithm to analyze segments of daily trajectories, we found evidence of specific repeating patterns in behavior within the home range of individual fish. Thus, *S. rivulatus* appears to have the ability to carry out its daily routines and revisit places of interest by employing sophisticated means of navigation while exploring its surroundings. In the future, using novel technologies for wireless recording from single cells of fish brains, *S. rivulatus* can emerge as an ideal system to study the neural basis of navigation in natural settings and lead to “electrophysiology in the wild.”

## Introduction

**FIGURE 1:**
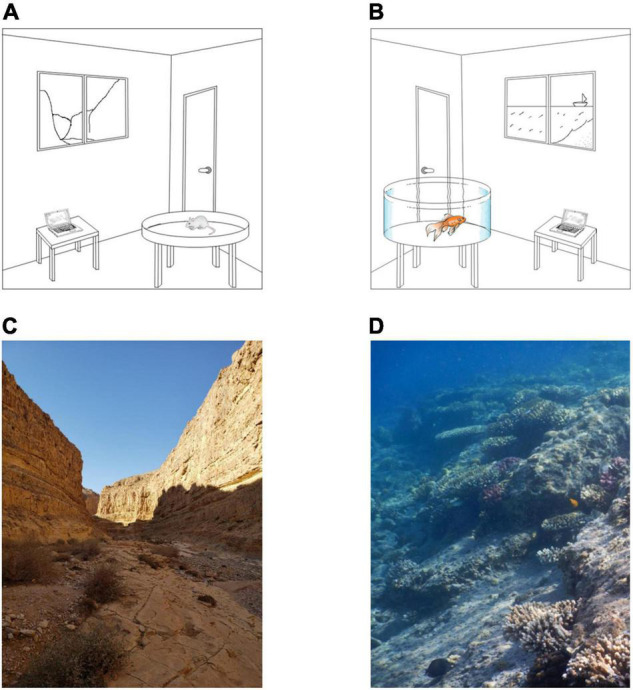
Lab versus natural environments. **(A,B)** Illustration of artificial lab environments for rodents and fish. The lab setting is restricted in terms of size, navigational cues, and complexity. **(C,D)** Two examples of natural environments for terrestrial and aquatic environments. The environments are limitless, rich in cues needed for navigation, and very complex.

To enable successful navigation, the brain must represent spatial information (Frost and Mouritsen, 2006; Mouritsen et al., 2016). Neural machinery has evolved to deal with the natural habitat and the ways in which different organisms move in and around it. However, almost all studies on navigation have been conducted in the lab where the navigated space is atypical, usually small, artificially enclosed, and sparsely occupied with artificial objects. In contrast, for many species, the natural habitat is open, lacks clear boundaries, is usually virtually limitless (relative to the animal), and is structurally complex (Tsoar et al., 2011; Jacobs and Menzel, 2014). For example, Figure 1 illustrates the small, restricted environments available in lab studies, as compared to vast complex natural environments. Since different spatial extents and complexities are likely to affect the internal representation of space, to unlock the full capacity of neural representations, the neural representation of the animal’s location and other parameters of space must be studied in the wild.

The exploration of neural representations *via* electrophysiology can teach us a great deal about the ways the brain collects and codes information about its surroundings. Research has identified different types of cells involved in the spatial coding of the surroundings. Cells coding different places, termed place cells or grid cells of more than one location coded with specific geometric attributes have been found in rodents (O’Keefe and Dostrovsky, 1971), bats (Yartsev et al., 2011; Yartsev and Ulanovsky, 2013), and primates (O’Mara et al., 1994; Furuya et al., 2014; Courellis et al., 2019). Cells coding for certain attributes of the environment, such as border cells and edge cells, have been documented in rodents (Solstad et al., 2008) and fish (Vinepinsky et al., 2020). Another type of cell activates in response to the animal’s behavior, and includes head direction cells in rodents (Taube et al., 1990), bats (Taube et al., 1990; Rubin et al., 2014; Ben-Yishay et al., 2021), and fish (Vinepinsky et al., 2020) as well as movement kinematic cells in rodents (Sargolini et al., 2006) and fish (Vinepinsky et al., 2020).

While natural environment is different from the small laboratory-based experiments, it is important to specify possible differences that will be obtained by extending the experimental effort outside. For example, animals might not represent the global position with respect to the environment (e.g., place cells), but instead represent the location with respect to a known route in the environment that the animal uses habitually and serves as a geographic anchor. In addition, place cells represent the location of the animal with place fields of about a 10 cm resolution in lab experiment of a 1 m arena. What happens when this space expands by a factor of 1,00,000? It might be that there are not enough cells in the brain to represent this space at the same resolution. The activity patterns of place and grid cells rely on repeating visits to the same locations in space. As the number of visits to every location in space decreases, what happens to the representation of space? Arenas in lab experiments are usually empty or contain only a few objects. A naturalistic environment can be large and populated with many objects. Does this affect the representation of spatial information?

Although electrophysiology in the wild can lead to a better understanding of the ways in which animals navigate in the real world, *in vivo* electrophysiology in the wild presents several difficult technological challenges. The first is the ability to obtain long-term *in vivo* recordings from a freely moving animal. The second is to do so while continuously and accurately monitoring the animal’s position and, possibly, its posture/behavior. This leads to a complex set of technological and experimental requirements that are likely to vary depending on the model animal. The recording technology needs to be both durable and equipped with a sufficiently large energy source, while being compatible with the size of the animal to not interfere with its freedom of movement while exploring the habitat. In terms of tracking equipment, none of the devices in the field should restrict the animal’s ability to explore the area. In addition to the technical difficulties, there are other challenges due to the natural environment. These include controlling for the numerous confounding variables in the natural environment such as predators, obstacles and other objects.

Clearly, a strategy to study fish requires implementing underwater electrophysiology. Although working underwater presents some difficulties with respect to water- and pressure-proofing the instruments, it does offer the advantage of making these instruments weightless (i.e., neutrally buoyant) and, thus permitting multi-day data acquisition. Even with the unavoidable need to adapt to the natural hydrodynamic form of the fish, prolonged data collection can be achieved with minimal interference with the animal’s behavior, a combination that may be unfeasible in many other model systems.

Recent studies have described a wireless electrophysiological system capable of recording the activity of single neurons in the brain of small free-swimming fish (Vinepinsky et al., 2017, 2020; Cohen et al., 2019). This system is based on a small data logger, which is mounted on the fish while it swims in the habitat. This technology can now support a 16-electrode array to record from the telencephalon, and specifically the pallium, of free-ranging fish and constitutes a critical step toward conducting naturalistic neuroscience.

While the technology described above can be used to monitor various brain-related capacities, its use constrains the selection of a model animal whose size, head shape and the durability of the skull make it possible to attach a neural logger. Ecological constraints exist as well, since if the fish tends to retreat or hunt in small spaces or relies on speed for its survival, the logger would put it at risk. In addition, it must be possible to attach an acoustic tag, which is needed to track the animal’s location; which further constrains the size of the study subject.

To study the neurobiology of free-range navigation, the model animal also needs to exhibit clear repetitive navigational patterns. This is of importance as it allows for future electrophysiological data to be collected numerous times from the same locations. In addition, the daily home range should be large enough to challenge the neural machinery to its full capacity, but not too large to enable continuous and accurate tracking of movements. The spatial features of these routines, as well as the habitat itself, should be non-trivial in terms of complexity, thus allowing informative patterns to emerge in the spatial distribution of the animal and the corresponding neural encoding.

Here we describe how these challenges can be met by using the unique advantages of a fish model. We argue that combining recently developed technology together with a careful selection of the fish species can lead to advances in our understanding of navigation. We perform the first step in characterizing the behavior of a possible fish model that can used for this study and show that the rabbitfish, *S. rivulatus*, meets these criteria as well as the constraints imposed by the technology.

For this purpose, we report on the spatial behavior of *S. rivulatus* in its natural reef habitat, as inferred using underwater acoustic telemetry. We describe the results obtained from the analyzes of daily trajectories of four fish, which were monitored for 14–60 days in the northern Gulf of Aqaba (Eilat) at the tip of the Red Sea. Consistent with earlier findings (Pickholtz et al., 2018), we found that the fish maintained a well-defined home range, including fixed sleeping sites. The fish adhered to predictable trajectories within the home range, and engaged in repeatable segments, forming patterns throughout the sections of these trajectories.

## Materials and Methods

### Ethics Statement

All experiments were approved by the Ben-Gurion University of the Negev Institutional Animal Care and Use Committee and were in accordance with government regulations of the State of Israel.

### Animals

**FIGURE 2:**
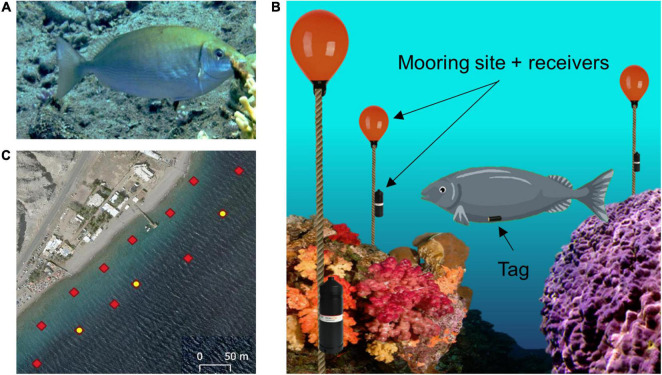
Experimental setup. **(A)** Profile view of a rivulated rabbitfish (*Siganus rivulatus*). **(B)** Illustration of the acoustic tracking system. The fish is fitted with an acoustic tag in the abdominal cavity. An array of acoustic receivers is attached to mooring sites spread throughout the reef environment. **(C)** Array layout deployed in the reef: red diamonds indicate each receiver station; the yellow dots indicate stations with an attached sync tag, which are used for synchronization of the clocks of all the receivers.

The model animal chosen for this study was the rivulated rabbitfish (*Siganus rivulatus*) as shown in Figure 2A. The study was conducted in its natural coral reef environment in the Gulf of Aqaba (Gulf of Eilat). *S. rivulatus* measuring 24–30 cm in body length and 150–190 g in body weight were used in this study. The fish were collected from the reef while scuba diving at night, to minimize stress to the fish. A small 5 g acoustic tag was surgically implanted in the peritoneal cavity according to the standard procedure (Bridger and Booth, 2003; Gehring et al., 2015; Pickholtz et al., 2018). After surgery, the fish were kept in a large tank of sea water for observation. They were returned to the reef after exhibiting clear signs of recovery, including normal swimming and foraging behavior.

### Acoustic Tracking of Fish Position

Underwater acoustic tracking technology (InnovaSea, Boston, MA, United States, and Thelma Biotel, Trondheim, Norway) was used to track the location of the fish over a long period of time. The implanted tags transmit an acoustic signal every 10 s which can be detected up to 70 m away and include the tag’s sensed depth and ID. The acoustic signals’ frequency range between 69 and 71 kHz, with varying power outputs of 140–150 dB, at these intensities and frequencies the fish are unaffected. The receivers are placed such that they form equilateral triangles for optimal coverage of the area. Once a transmission has been detected, the receivers log the transferred data and the transmission time of arrival. If three or more receivers detect the same transmission, the origin of the signal can be determined by triangulation. Figure 2B illustrates the underwater array, tagged fish and receiver layout. Several synchronization tags are positioned in the array to correct for the lag in synchronization between the different receivers. Given that the synchronization tags are located at known static locations, a comparison of their time of arrival with the actual logged ones can be used to adjust the clocks on the receivers. A bird’s-eye view of the array along with the locations of the synchronization tags is presented in Figure 2C.

To detect the signal’s origin location, two localization algorithms were used. The first, solving an optimization problem. For every couple of signal detecting receivers, the difference in time of arrival (DToA) can be calculated. With the DToA, a 3-Dimentional map of the reef detailing the DToA error from every location is created. Such maps, created for every receiver couple combination, are overlapped to find a single location with minimal error.

The second algorithm used machine learning to calculate positions based on the time of arrival of the tag transmissions and ground truth data. Each tag transmits a signal with a distinct identifier (tag ID) detected by geo-referenced underwater receivers distributed in the study area, and tag localization is computed using millisecond-scale differences in signal time-of-arrival to each receiver. In order to estimate localization errors, we ran ground-truth tests with a GPS device (Montana 680t, Garmin, Olathe, KS, United States) within and at the perimeters of the experimental array, at different times during the study. The median localization error was established as 4 m. Ground-truth data collection was performed by snorkelers or divers moving across the array with several acoustic tags (InnovaSea and Thelma Biotel) at varying depths (0–15 m).

### Data Analysis

#### Definition of the Home Range

To define the home range, we calculated a set of locations by using the alpha shape algorithm with a shrink factor of 0.5 (Matlab boundary function) around the data points of the fish trajectories. The results of the alpha shape algorithm were compared to the kernel-based method (Seaman and Powell, 1996) and found to be similar.

#### Occupancy Map

The home range was split into 5 m × 5 m bins. For each bin the time spent by each fish was calculated. The map was filtered with Gaussian filter (σ = 2.5 m). A base-10 logarithm was used for visualization using heatmap.

#### Detecting Patterns in Fish Daily Trajectories

To detect repeated patterns of swimming, we linearly interpolated the trajectories to obtain a 1-min interval resolution. Interpolation resulted in daily trajectories of equal time resolution.

#### Data Quality Control

The acoustic tracking cannot follow the fish continuously since occasionally the cluttered acoustic environment blocks transmissions from the acoustic tag or occludes the acoustic signal. This type of disruption is more common during the night since the fish sleep on the sea floor. To ensure data quality for this part of the analysis, we selected contiguous days that had a minimum of 30 data points per hour. We allowed for larger data gaps when analyzing the home range and establishing the sleeping grounds.

#### Segmentation of Trajectories

To overcome the inherent difficulty in classifying different types of behavior based on long swimming trajectories, we segmented the daily trajectories into 30-min-long segments according to the approach presented in Gehring et al. (2015). In this approach, the segments are used to classify different behaviors based on repetitive attributes of the segments. For this purpose, the daily trajectory was defined as the time between sunrise and sunset in Eilat, with adjustments for seasonal differences between fish. At first, each daily trajectory was segmented into 30-min-long paths with a 50% overlap to avoid phase related phenomena. This was adjusted in later analyzes when no significant phenomena occurred.

#### Computation of Path Features

Prior to classification, we calculated a set of 13 features that quantified different geometric and temporal properties of the trajectory segments, as listed in Table 1. The features were adapted to our data from a set of features defined in previous studies (Gehring et al., 2015; McLean and Skowron Volponi, 2018). Features were divided by the standard deviation of each feature to derive a unit-free measurement.

**TABLE 1:** Features characterizing the geometric and temporal aspects of trajectory segments.

Feature	Definition
Distance	$D=\left|\vec{x_{n}}-\vec{x_{i}}\right|$, where $\vec{x_{i}},\vec{x_{n}}$ are the first and last locations in the segment
Length	$L=\sum_{i=1}^{n-1}\left|\vec{x_{i+1}}-\vec{x_{i}}\right|$, where $\vec{x_{i}}$ are locations within the segment
Straightness	$S=\frac{D}{L}$
Area	A, Area of convex hull enclosing segment
Median speed	$M\,S=m\,e\,d\,i\,a\,n\,\left(\vec{S}\right),\vec{S}=\left[\frac{\left|\vec{x_{2}}-\vec{x_{1}}\right|}{1\,m},\ldots ,\frac{\left|\vec{x_{n}}-\vec{x_{n-1}}\right|}{1\,m}\right]$
Mean distance from center	ρ, distance of segment center of mass from the median of entire data set
Direction auto correlation 5 min	DAC5, correlation coefficient of the swimming directions of 5-min part segments
Direction auto correlation 10 min	DAC10, correlation coefficient of the swimming directions of 10-min part segments
Speed auto correlation 5 min	SAC5, correlation coefficient of the swimming speed of 5-min part segments
Speed auto correlation 10 min	SAC10, correlation coefficient of the swimming speed of 5-min part segments
Day phase	DP, normalized time of segment in the day
Mean angle from center	θ, cosine of the angle between segment center of mass and X axis
Focus	$F=1-\frac{4\,A}{\pi \,L^{2}}$

*Formal definition of the segment features ranging from fundamental features (e.g., length) to derived features (e.g., focus). All features were normalized to obtain dimensionless features for the clustering algorithm.*

#### Classification of Segments Into Classes

To detect repeated patterns of fish movement, we used a semi-automated clustering algorithm. We first calculated a set of features for each segment that described the geometry, kinematics and temporal characteristics of the segments followed by a standard clustering algorithm as described in detail below.

#### Dimensionality Reduction

We used principal-component analysis of the 13 features to isolate a limited number of independent dimensions that described the trajectory segments. Based on the eigenvalues, we selected the first four components, which explained 67% of the variance, as the basis of the clustering algorithm.

#### Semi-Supervised Clustering

To find classes of trajectory segments in each fish, we applied the following clustering algorithm to the first four principal components. We applied agglomerative clustering to obtain an initial set of classes. This was done using the Euclidian distance as a similarity measure (Matlab cluster function). The number of classes was determined by examination of the merger score as clusters merged at each step, and finding the step at which the differences between clusters exceeded 1.5%. Different thresholds were tested with no significant impact on the results. After automatic clustering, the classes were observed and labeled.

## Results

We analyzed the patterns of trajectories of the *S. rivulatus* to establish this species as a model animal for the study of the neural basis of spatial cognition. For this purpose, we recorded the trajectories of ten fish in their natural coral reef environment, along the north-eastern shoreline of the gulf of Aqaba (Eilat) in the Red Sea. Fish were tracked for periods of 14–60 days.

### *Siganus rivulatus* Maintain a Home Range in the Reef Environment

**FIGURE 3:**
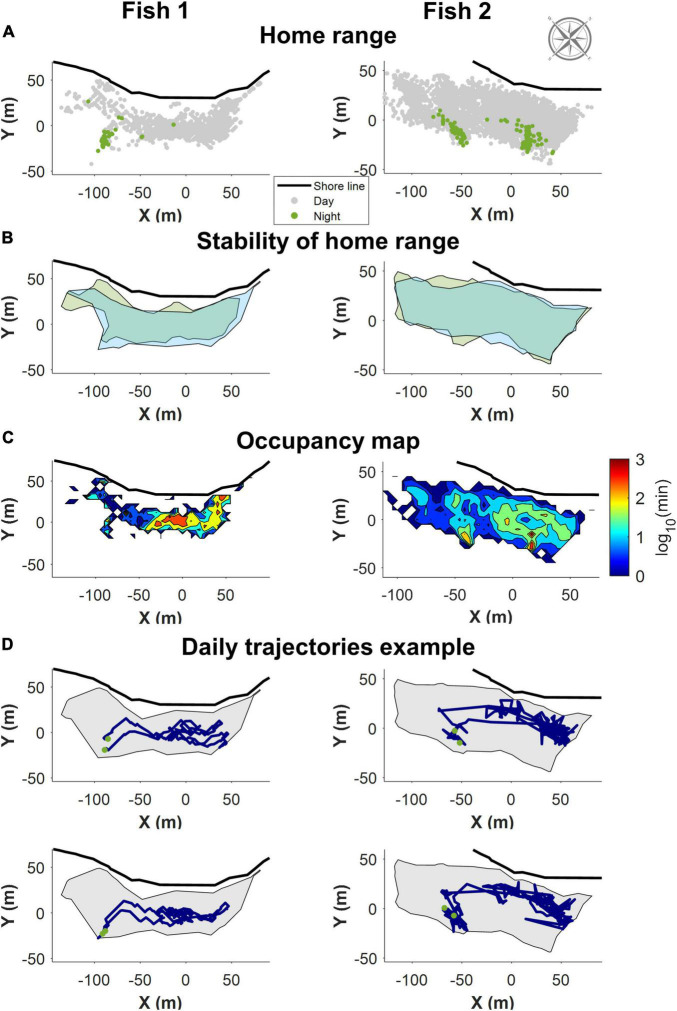
Example of the home range trajectory characteristics of two fish with data obtained through the first of the positioning algorithms. All figures are shown along with the relevant section of shoreline and compass. **(A)** All recorded positions for the fish. In gray are the positions during the day, in green the night-time position. The fish slept in a restricted area in the home range. **(B)** The home range remained stable over time. Boundaries of the area covered the first few days of data in green, and the last few days in blue. **(C)** A heat map visualizing a normalized logarithmic scale of the occupancy map in different areas of the home range. **(D)** Two examples of daily trajectories for each fish. Green dots represent the start and end points for each day. The gray background is the boundary delineating the home range.

Consistent with previous observations (Pickholtz et al., 2018), in the northern Gulf of Aqaba (Eilat), *S. rivulatus* concentrated their daily activity within a home range of approximately 200 m × 50 m, as shown in Figure 3A. The home range remained stable over time as shown in Figure 3B, where the areas covered by the fish in the first and last days of the study are marked. Within the home range, individual fish visited some areas more frequently than others (Figure 3C). These areas corresponded to locations the fish passed through regularly and locations where they spent their time foraging. The fish returned to the same sleeping sites (i.e., sites utilized between sunset and sunrise), with high regularity, as exemplified in Figure 3C. An examination of the daily trajectories showed that the fish spent much of the day away from the sleeping site (e.g., in Figure 3D).

### *Siganus rivulatus* Exhibits Multiple Strategies of Environment Exploration

**FIGURE 4:**
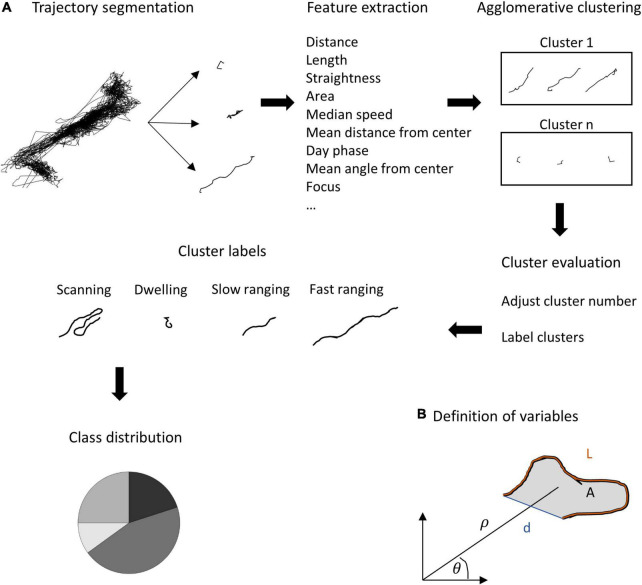
Feature calculations and semi-supervised clustering algorithm. **(A)** The daily trajectory was segmented into overlapping segments. For each segment, a set of features was calculated (see Table 1 for formal definitions). Agglomerative clustering was applied to the data to obtain a set of classes of trajectory segments. Finally, a manual correction for similar classes was applied to avoid over- splitting of the classes. **(B)** Definition of the fundamental features used for clustering: the path length of the segment (L), the total area of the segment which was defined as the polygon covering the set of points of the segment, the distance from the segment’s start and end, (d) The distance and direction of the center of mass of all points defining the segment from the center of the trajectory (ρ and θ, respectively).

Since elementary measures such as the home range size or occupancy cannot capture the full extent of fish behavior, we looked for repeated patterns in the swimming trajectories of the fish. For this purpose, we segmented the daily trajectories into overlapping 30 min segments (see section “Material and Methods”, Analysis). Then we extracted spatial and temporal features from each segment (Table 1 and Figure 4), followed by principal component analysis for dimensionality reduction. Finally, we used a semi-automatic clustering to detect classes of trajectory segments (see section “Materials and Methods”).

**FIGURE 5:**
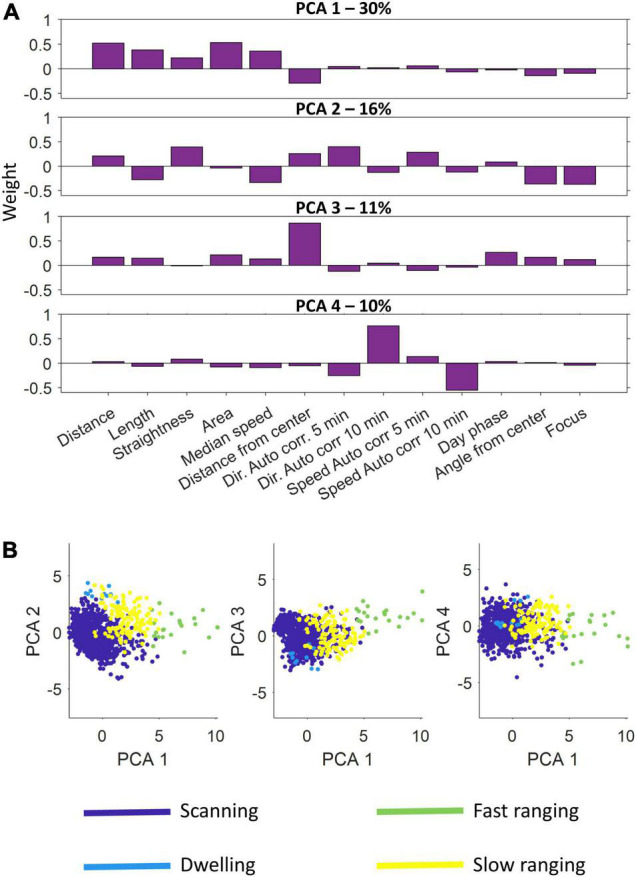
Principal component analysis and semi-automatic agglomerative clustering. **(A)** A principal component analysis was performed on the feature sets of the segments. Shown are the four principal components used. Each component emphasizes a different set of features. **(B)** After dimension reduction using principal component analysis, semi-automatic agglomerative clustering was performed to divide the segments into four classes: scanning, dwelling, fast ranging and slow ranging.

Figure 5 presents the results of the principal component and clustering analyzes for one fish using data obtained through the second of the positioning algorithms. The four principal components varied in score assigned to each feature (Figure 5A). Principal component 1 had a higher score to the distance and area features, a negative score to the distance from the center and a near neutral to all others. In contrast, principal component 3 was nearly neutral for all features except the distance from the center to which it had a high score. In a similar manner principal component 4 focused on two specific features, the autocorrelation for direction and speed, to which it had a high score and a negative score, respectively. Principal component 2 accounted for a variety of features both positively and negatively. The four principal components explained 30, 16, 11, and 10% of the variance, respectively.

**FIGURE 6:**
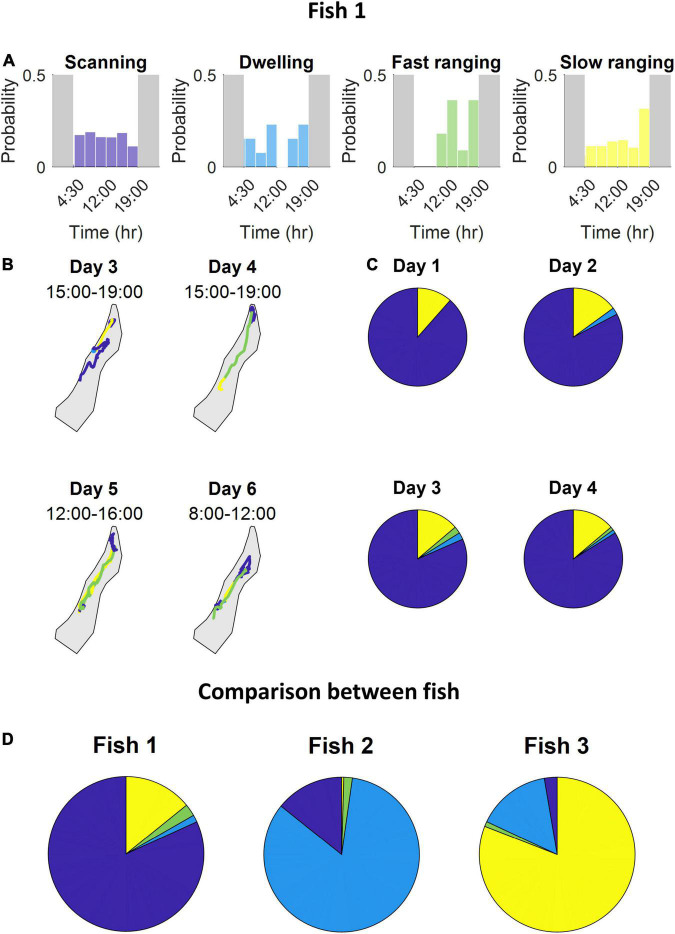
The fish use multiple strategies during the day while exploring the coral reef environment. **(A)** For each class, the probability segments appearing at specific times throughout the day. **(B)** Several examples of 4-h long trajectory segment sequences the fish used to travel between areas where it scans and dwells. **(C)** The time one fish spent engaging in each class of trajectory segments over four consecutive days. The days were similar in their class distribution. **(D)** Time-division of engagement in each class throughout the entire tracking period of each fish. Stability tested among different day groups resulted in a standard error percentage of fish 1 – [0.2,23,36,2.4], fish 2 – [22,4.2,71,0], fish 3 – (28,0.6,24,0.4) for scanning, dwelling, fast ranging and slow ranging, respectively. Variation tested among all fish yielded that all but one class have a substantial variability with a minimal standard error of 75%, fast ranging being the exception with a standard error of 24% (all color maps relate to Figure 5B).

After scoring all the segments according to the four principal components, the agglomerative clustering algorithm was run and the classes were defined (Figure 5B and see section “Materials and Methods”). We found that the *S. rivulatus* exhibited four main trajectory segment patterns. We defined these four trajectory segment classes as follows: (1) scanning: the fish scans a small part of the home range; (2) dwelling: the fish spends time without moving; (3) long ranging: the fish travels long distances during the segment; and (4) short ranging: the fish travels short distances during the segment. Figure 6 presents several examples of the trajectory segment analysis for fish 1. Several examples, taken at random times during the tracking period, of 4-h long trajectory segment sequences for one fish are presented in Figure 6B. Here, ranging, both slow and fast, were interspersed between periods of scanning and dwelling.

Inspection of the behavior across fish, throughout the entire tracking period, revealed that different fish varied in terms of each class (Figure 6D). Some fish spent most of the daily trajectory in the scanning class while other fish spent most of the daily trajectory in the dwelling or slow ranging classes. In addition, the fish were consistent in their different trajectory patterns. This can be seen in the occupancy of the trajectory segment classes across days (Figure 6C).

## Discussion

This study was designed as a preliminary investigation toward establishing the *S. rivulatus* as an animal model for the study of the neural basis of navigation in the wild. For this purpose, we measured the locations of fish in their natural coral reef environment. We characterized the trajectory patterns in the natural environment. We found that the fish maintained a home range, the area in which the animal lives and moves on a regular basis (Figure 3). The home range was relatively stable over time. Overall, the *S. rivulatus* behavior was similar with other coral reef fish which maintain home range (Kramer and Chapman, 1999). The fish also returned to roughly the same place to sleep every night. This is an indication that *S. rivulatus* can plan daily routes that start and end in the same place.

We found that the trajectory segments fell into four different classes (Figure 5B). This is an indication that the fish used multiple movement patterns in the coral reef environment. Each fish had individual ratios of using the different four strategies of movement (Figure 6D) and these different ratios were relatively stable across days for each fish with (Figure 6C) higher variability for the lower ratio used strategies. Overall, our results indicate that *S. rivulatus* evidences a complex spatial pattern of movement in the environment.

This work extends the findings of two previous works on the rabbitfish family. The first, a study of *S. rivulatus*, demonstrated the existence of home range behavior in this species in two different environments: The Red Sea and the Mediterranean. The behavior of the fish in the two environments were different in scale, since the fish maintained a much larger home range in the Mediterranean possibly due to substantial differences in the distribution of food in this environment (Pickholtz et al., 2018). The second study found that juvenile rabbitfish (*S. corallinus and S. doliatus*) defined a home range that could be relatively small, and also showed strong homing behavior (Ghanawi et al., 2013; Givon et al., 2022). Our findings thus contribute to a better understanding of the spatial behavior of this species by providing a detailed analysis of the movement strategies employed by the fish in their natural environment.

More broadly in the field of fish navigation there are indications that homing salmons use different strategies which rely on different sensory modalities to return to their spawning grounds (Quinn and Brannon, 1982; Dittman and Quinn, 1996). Here, we showed that *S. rivulatus* use different patterns while exploring the coral reef environment reflected in the trajectory segments classes. In this respect the *S. rivulatus* is similar to the salmon in terms of using different behaviors during exploration. In addition, the fish were shown to use different egocentric (Rodríguez et al., 2002), geometric (Sovrano et al., 2002; Broglio et al., 2011) and visual (Rodríguez et al., 2002; Givon et al., 2022) cues when navigating in different lab settings. In our study, we showed that the trajectory segments could be classified into classes. It remains to be seen which sensory modalities and environmental cues are used by these animals in their daily routes.

In future works it will be possible to add a layer of an electrophysiological aspect to that of the tracking. This raise concerns on the issue of tracking resolution and fish recapturing.

First concern is whether the tracking resolution high enough to allow correlating location and brain activity. This is dependent on resources mainly, with the deploying more receivers in the field the positioning error can be reduced to about a meter. Furthermore, new technologies, including robotics that follow the fish using both acoustic and visual sensors are in development (Zolich et al., 2017). Such robotics can provide more accurate monitoring of fish location and additional dynamics parameters such as head direction and even feeding and social interaction with other conspecific.

The second issue that needs to be addressed is the need to recapture the fish in order to obtain the electrophysiology data. As currently, the technology for real-time positioning exists and relies on acoustic receivers that transmit detection continuously. This allows detection of the fish location at night while the fish sleeps and allows recapturing. All these technologies can provide the needed additional capacity to allow electrophysiology in the wild.

### Outlook for Naturalistic Neuroscience

*Siganus rivulatus* and the coral reef environment both emerged as excellent choices for studying the neural coding of spatial information in the wild. These fish are excellent navigators and can accurately find their way in the coral reef environment. While the size of the natural home range of *S. rivulatus* in the Gulf of Eilat is qualitatively larger than typical lab settings (approx. 200 m × 50 m × 5 m vs. 1 m × 1 m × 1 m), it is still relatively small compared to other species. Thus, with proper equipment it remains tractable in terms of our ability to track the fish (both visually and acoustically), its behavior, and its immediate surroundings. The coral reef habitat of this species represents a non-trivial environment characterized by high complexity and rich content. The elongated topography of the Gulf of Aqaba and its coral reef constrains navigational routes to a pseudo-linear structure, potentially simplifying the neural representation (at the habitat scale) and data analysis. Finally, the capacity to control buoyancy in the aquatic environment makes it possible to fit the fish with a neurological data logger with enough battery power to record brain activity continuously for a week.

The main technological breakthrough that enables the next step of electrophysiology in the wild is the recent development of wireless recording system from behaving fish (Vinepinsky et al., 2017, 2020; Cohen et al., 2019). This technology, which weighs only 2.5 g, enables continuous recording from a fish for several days. This is achieved, in part, by adjusting the floatation of the device so that it is neutrally buoyant such that a 250 g fish can be fitted with a relatively large 35 g battery. This type of experiment means that weeklong data can be collected in a single experiment. With proper design, the neutral buoyancy implant neither interferes with the fish’s ability to maintain its balance nor increases drag in a significant way. In experiments with goldfish, the fish were able to swim with this device for up to 2 weeks without any behavioral effects whatsoever (Vinepinsky et al., 2017, 2020; Cohen et al., 2019). This neural logger technology can be modified to record from the brains of fish swimming in the wild and would enable significant insights into the neural basis of navigation in the real world. It is important to note that the home range size of the *S. rivulatus* is also make it possible to retrieve the data logger device since at the end of the experiment using acoustic tag.

From lesion studies in the goldfish (Rodríguez et al., 2002), it is known that the relevant region in the brain is the later pallium at the telencephalon and recording should be targeted in this region. Another option is to record from the central region of the telencephalon that contain cell bodies with connections in other regions of the telencephalon (Northcutt, 2008). In summary, this is an indication that 16 electrodes array is of sufficient resolution to obtain indication about representation of space in the rabbitfish brain.

It should be noted that *S. rivulatus* is not the only choice for the development of electrophysiology in the wild. Other fish species with similar home range sizes can serve a similar purpose, including marine (Kramer and Chapman, 1999) and freshwater species (Baktoft et al., 2017). Beyond fish, the Egyptian bat was developed in recent years into an animal model to assess spatial cognition in large linear arenas (Eliav et al., 2021) and is expected to provide further insights in the future.

Overall, we showed that *S. rivulatus* maintains a home range and can navigate in its natural coral reef environment. The fish can start and end their daily trajectory in roughly the same place. In addition, the fish can use different navigational strategies. The size of the home range is very large compared to regular lab experiments but still small enough to make tracking the fish feasible. The combination of these properties with the technology of recording single cells in the fish brain can lead to the development of electrophysiology in the wild and can provide insights into the neural basis of navigation.

## Data Availability Statement

The raw data supporting the conclusions of this article will be made available by the authors upon reasonable request.

## Ethics Statement

The animal study was reviewed and approved by the Ben-Gurion University of the Negev Institutional Animal Care and Use Committee.

## Author Contributions

SG: formal analysis, investigation, and methodology. RP: investigation. EP: methodology. OB-S, MK, and RS: conceptualization and investigation. All authors contributed to the article and approved the submitted version.

## Conflict of Interest

The authors declare that the research was conducted in the absence of any commercial or financial relationships that could be construed as a potential conflict of interest.

## Publisher’s Note

All claims expressed in this article are solely those of the authors and do not necessarily represent those of their affiliated organizations, or those of the publisher, the editors and the reviewers. Any product that may be evaluated in this article, or claim that may be made by its manufacturer, is not guaranteed or endorsed by the publisher.
